# Cost-Sharing Disparities for Out-of-Network Care for Adults With Behavioral Health Conditions

**DOI:** 10.1001/jamanetworkopen.2019.14554

**Published:** 2019-11-06

**Authors:** Wendy Yi Xu, Chi Song, Yiting Li, Sheldon Michael Retchin

**Affiliations:** 1 Division of Health Services Management and Policy, College of Public Health, The Ohio State University, Columbus, Ohio; 2 Division of General Internal Medicine, Department of Internal Medicine, College of Medicine, The Ohio State University, Columbus, Ohio; 3 Division of Biostatistics, College of Public Health, The Ohio State University, Columbus, Ohio

## Abstract

**Importance:**

Individuals in the United States with mental illnesses and substance use disorders can face major access barriers from limited provider (eg, clinicians and facilities) networks in health insurance plans.

**Objective:**

To evaluate the cost-sharing payments for out-of-network (OON) care for private insurance plan enrollees with mental health conditions, alcohol use disorders, or drug use disorders compared with those with congestive heart failure (CHF) or diabetes.

**Design, Setting, and Participants:**

This cross-sectional study used data from a large commercial claims database from 2012 to 2017. The study included adults with mental health conditions, with alcohol use disorders, with drug use disorders, with CHF, and with diabetes who were aged 18 to 64 years and enrolled in employer-sponsored insurance plans.

**Main Outcomes and Measures:**

Main outcomes included OON care during hospitalization, OON care during outpatient care, cost-sharing payments with OON care, OON cost sharing as a proportion of total health care spending, and OON cost sharing as a proportion of total cost sharing.

**Results:**

The study sample included 3 209 929 enrollees with mental health conditions (mean [SD] age, 45.9 [12.6] years; 64.8% women), 294 550 with alcohol use disorders (mean [SD] age, 42.8 [13.4] years; 60.9% men), 321 535 with drug use disorders (mean [SD] age, 41.1 [13.9] years; 59.1% men), 178 701 with CHF (mean [SD] age, 53.8 [8.9] years; 62.6% men), and 1 383 398 with diabetes (mean [SD] age, 52.5 [9.0] years; 58.9% men). Enrollees with behavioral conditions were more likely to encounter OON clinicians in inpatient and outpatient settings. For instance, those with drug use disorders were 12.9 percentage points (95% CI, 12.5-13.2 percentage points; *P* < .001) more likely to have inpatient OON care than those with CHF and 15.3 percentage points (95% CI, 15.1-15.6 percentage points; *P* < .001) more likely to receive outpatient OON care. Behavioral conditions also had higher cost sharing for OON care. For example, individuals with mental health conditions had cost-sharing payments for OON care $341 (95% CI, $331-$351) higher than those with diabetes (*P* < .001), individuals with drug use disorders had cost-sharing payments for OON care $1242 (95% CI, $1209-$1276) higher than those with diabetes (*P* < .001), and individuals with alcohol use disorders had cost-sharing payments for OON care $1138 (95% CI, $1101-$1174) higher than those with diabetes (*P* < .001). The OON care rates and cost-sharing payments were much higher when enrollees sought care from behavioral clinicians and facilities.

**Conclusions and Relevance:**

In this cross-sectional study of enrollees in commercial insurance plans, cost sharing for OON care among those with behavioral health conditions was significantly higher than those with chronic physical conditions. These disparities may be indicative of limited in-network availability for behavioral health care.

## Key Points

**Question:**

What are the differences in out-of-network care for private insurance plan enrollees with behavioral health conditions compared with enrollees with chronic physical conditions?

**Findings:**

In this cross-sectional study, analyses using a large commercial claims database indicated that the cost sharing from out-of-network care among those with behavioral health conditions was significantly higher than payments for those with other prevalent chronic physical conditions. Among those with behavioral health conditions, rates of out-of-network care were higher for behavioral clinicians and facilities than for nonbehavioral sources.

**Meaning:**

Despite parity laws, disparities in out-of-network care and cost sharing for those with behavioral health conditions may create undue financial burdens and reflect access-to-care barriers.

## Teaser

This cross-sectional study evaluates the cost-sharing payments for out-of-network care for insurance plan enrollees with mental health conditions, alcohol use disorders, or drug use disorders compared with those with congestive heart failure or diabetes.

## Introduction

Nearly 57 million US adults have behavioral health conditions, such as mental illnesses or substance use disorders.^1^ These individuals also frequently have higher risks of poor physical health, with a wide range of chronic physical conditions.^2^ Among those with behavioral health conditions with unmet health care needs, access to available health care providers (eg, clinicians and facilities) and costs represent major barriers to care.^3^ Tackling these barriers has been a long-standing focus of policy makers. The Paul Wellstone and Pete Domenici Mental Health Parity and Addiction Equity Act of 2008 was intended to address the parity of benefits—private health plans are not allowed to impose higher cost-sharing requirements or other restrictions (eg, number of visits) for mental health or substance use disorders than requirements for medical and surgical benefits.^4^

However, access to timely care can still be challenging for patients in private health plans who have behavioral conditions, especially when narrow networks include an insufficient number of specialists.^5,6,7,8^ Many patients with behavioral conditions need multiple clinicians, including both behavioral and nonbehavioral clinicians. However, privately insured adults with mental disorders have reported greater difficulty accessing appropriate health care compared with those without behavioral conditions.^9^

Clinicians and facilities that decline to accept price discounts for participation in health plan networks are considered out of network (OON). Recent evidence has indicated a prevalent use of OON clinicians and facilities among privately insured enrollees.^10^ Although the parity law has improved access to OON care for patients covered by private insurance,^11^ obtaining care from OON providers can come with a price. Steeper cost-sharing payments, such as higher deductibles and higher coinsurance rates, are typically required for care from OON providers.^12^ Although the maximum annual out-of-pocket cost sharing in private plans is capped under the Patient Protection and Affordable Care Act, this cap applies only to in-network health care.

Even with private insurance, individuals with behavioral health conditions may not be able to afford the higher costs from OON providers; in addition, steeper out-of-pocket expenses may further constrain patients’ access to needed behavioral care. Therefore, we evaluated the cost-sharing payments for OON care for enrollees with mental health conditions, alcohol use disorders, or drug use disorders. We contrasted these with enrollees having other highly prevalent chronic physical conditions, congestive heart failure (CHF) and diabetes.

## Methods

### Data Source and Study Sample

In this cross-sectional study, we pooled 2012-2017 data from the Truven Health MarketScan Commercial Claims and Encounters Database, a nationwide insurance claims database that includes detailed information regarding treatment episodes, such as detailed diagnoses, procedures, and care settings. The data also indicate whether clinicians and facilities were in a patient’s insurance network and contain actual reimbursements based on network status. The Ohio State University Institutional Review Board exempted this study from review because we used deidentified secondary data that do not involve human participants. This study followed the Strengthening the Reporting of Observational Studies in Epidemiology (STROBE) reporting guideline.

We identified all adults aged 18 to 64 years who enrolled in employer-sponsored insurance (ESI) plans as policy holders or dependents. These ESI enrollees were primarily from plans sponsored by large employers. Individuals who did not incur any covered health care expenses were excluded from the study (12% of all ESI enrollees). Our study also required individuals in the analysis to be continuously enrolled for at least 1 full calendar year with medical and prescription drug coverage and required that data be available to determine payments for OON care.

We used claims-based algorithms established by the Centers for Medicare & Medicaid Services Chronic Condition Data Warehouse^13^ to identify individuals with mental health conditions (depression, personality disorders, anxiety disorders, bipolar disorders, schizophrenia, or posttraumatic stress disorder), alcohol use disorders, and drug use disorders. The Chronic Condition Data Warehouse algorithms use 2-year reference windows. Thus, those with the qualifying diagnoses within any month of the current or an immediate previous calendar year were included. For comparison, we identified individuals with 2 common chronic physical conditions, CHF and diabetes, also using the Chronic Condition Data Warehouse algorithms. We excluded enrollees with co-occurring behavioral conditions from the CHF and diabetes samples.

### Outcome Measures

We measured the following 5 outcomes for each sample in the study: (1) whether or not an individual received OON care for inpatient care; (2) whether or not an individual received OON care for outpatient care; (3) the cost-sharing payments associated with OON care, including copayments, coinsurance, and deductibles; (4) cost sharing for OON care as a proportion of total health care spending, where the total spending included all payments by insurance plans and cost-sharing payments; and (5) cost sharing for OON care as a proportion of total cost sharing (ie, for both in-network and OON care). The spending measures were adjusted to 2017 dollars. Because services paid entirely out of pocket were not considered insurance benefits and were missing in the claims data, these expenses were not included in this study. All outcome measures were calculated per person per year.

For those with behavioral conditions, we further evaluated the specific use of OON behavioral facilities and clinicians, in both inpatient and outpatient settings. Behavioral facilities included mental health or chemical dependence treatment centers and mental health facilities, as well as mental health outpatient day treatment programs. The behavioral care clinicians included psychiatrists, psychiatric nurses, and psychologists. Specifically, for each individual, we calculated the proportion of OON claims for each provider type divided by the total number of claims (in network and OON) for outpatient behavioral facilities, outpatient nonbehavioral facilities, outpatient behavioral clinicians, outpatient nonbehavioral clinicians, inpatient behavioral facilities, inpatient nonbehavioral facilities, inpatient behavioral clinicians, and inpatient nonbehavioral clinicians. For each category, we also measured the cost-sharing payments for OON care. The OON claims that resulted from OON laboratories or other supply centers were not included in these measures.

### Statistical Analysis

The descriptive statistics summarized the distribution of outcome measures in each sample. We further modeled the marginal effects of having a behavioral condition on each outcome measure relative to individuals with CHF or diabetes,^14^ adjusted for individual-level variables to account for differences that could be associated with spending and health care use. A separate logistic regression model was used to estimate the probability of OON care for individuals in each behavioral condition group relative to CHF or diabetes. A generalized linear regression model with log link and γ distribution was fitted to estimate the expected OON cost-sharing payments. Another generalized linear regression model with binomial distribution and log link was used to estimate the expected cost-sharing proportions for OON care.^15,16^ Robust SEs by individual enrollees accounted for the fact that an individual could be observed multiple times during the study interval. All *P* values were from 2-sided tests and results were deemed statistically significant at *P* < .05. Stata, version 14 (StataCorp) was used for analysis.

The models controlled for enrollee plan characteristics as reflected by plan types, including comprehensive plans, health maintenance organizations, exclusive provider organizations, preferred provider organizations, point-of-service plans, and high-deductible and consumer-driven health plans. We adjusted for demographic and clinical characteristics using the Department of Health and Human Services Hierarchical Condition Categories risk adjustment model developed for commercially insured populations.^17^ A risk score, which took into account age and sex as well as health conditions associated with diagnoses in a year, was assigned to each enrollee. Both *International Classification of Diseases, Ninth Revision,* and *International Statistical Classification of Diseases and Related Health Problems, Tenth Revision* codes were used to construct the scores. The risk scores are a proxy for health status, with higher risk scores indicating more severe illness and more complex health care needs. Because the Department of Health and Human Services Hierarchical Condition Categories risk scores accounted for age and sex, the regression model did not separately adjust for these 2 factors. Because access to in-network providers may differ between rural and urban areas, we also considered rural residency. Our models also controlled for state fixed effects to account for differences in state policies that potentially affected OON payments and provider network adequacy.^18,19^ The detailed model specifications are included in the eAppendix in the Supplement.

Finally, data on the observed ESI enrollees in our sample were weighted to reflect the national population of individuals with ESI by demographic strata. Sampling weights were constructed using the Public Use Microdata Sample of the American Community Survey conducted by the US Census Bureau.^20,21^ The weights were considered in the regression analysis.

Although the OON experiences from enrollees with behavioral conditions were contrasted with those with other chronic physical conditions, the analyses were not meant to be interpreted as causal inferences. Because of a cross-sectional design, the results may only capture the outcomes at a given time. Moreover, the observed results may show both actual differences between groups in comparisons and the unobserved clinical information that affected the OON care outcomes.

## Results

As shown in Table 1,^17^ the samples included 3 209 929 enrollees with mental health conditions (mean [SD] age, 45.9 [12.6] years; 64.8% women), 294 550 enrollees with alcohol use disorders (mean [SD] age, 42.8 [13.4] years; 60.9% men), 321 535 enrollees with drug use disorders (mean [SD] age, 41.1 [13.9] years; 59.1% men), 178 701 enrollees with CHF (mean [SD] age, 53.8 [8.9] years; 62.6% men), and 1 383 398 enrollees with diabetes (mean [SD] age, 52.5 [9.0] years; 58.9% men). The person-year observation numbers are also shown in Table 1.^17^ The mean (SD) age was 45.9 (12.6) years for those with mental health conditions, 42.9 (13.4) years for those with alcohol use disorders, 41.1 (13.9) years for those with drug use disorders, 53.8 (8.9) years for those with CHF, and 52.5 (9.0) years for those with diabetes. Most enrollees were male, except that 64.8% of those with mental health conditions were female. Overall, most individuals lived in metropolitan areas. The plan-type distribution in our analysis was similar to the distribution observed in other large employer-sponsored plans.^22^ Adults with CHF had the highest Hierarchical Condition Categories scores, reflecting more complex and intensive health care needs.

**Table 1.** Study Sample Characteristics^a^^,^^b^

Characteristic	Mental Health Conditions	Alcohol Use Disorders	Drug Use Disorders	Congestive Heart Failure^c^	Diabetes^c^
Age, mean (SD), y	45.9 (12.6)	42.9 (13.4)	41.1 (13.9)	53.8 (8.9)	52.5 (9.0)
Female, %	64.8	39.1	40.9	37.4	41.1
Rural residency, %	10.4	9.2	9.2	14.2	13.5
HCC score, by percentile, mean (SD)^d^	2.8 (6.8)	4.6 (10.1)	4.9 (10.3)	12.2 (16.5)	3.7 (7.0)
25th	0.5	0.5	0.5	4.0	1.6
50th	0.7	1.6	1.8	5.9	1.8
75th	2.3	4.1	4.8	12.6	2.4
Insurance plan type, %					
HMO	11.7	15.7	15.4	9.4	11.5
PPO	55.3	50.6	51.1	56.9	56.3
HDHP	22.0	21.6	21.3	20.3	20.2
EPO	0.8	0.8	0.8	1.0	1.0
POS	7.1	6.5	6.6	7.7	7.4
Comprehensive plan	3.3	4.8	4.8	4.8	3.8
Total health care spending, mean (SD), $^e^	15 067 (42 272)	25 300 (59 332)	27 478 (63 541)	44 369 (99 642)	14 578 (40 290)
Percentile					
25th	1912	2451	2681	4118	1932
50th	4932	8233	9224	13 079	5374
75th	13 255	24 234	26 683	43 431	13 017
No. of enrollees	3 209 929	294 550	321 535	178 701	1 383 398
No. of person-year observations	6 127 034	492 358	536 224	331 745	3 478 181

Abbreviations: EPO, exclusive provider organization; HCC, Hierarchical Condition Categories; HDHP, high-deductible and consumer-driven health plan; HMO, health maintenance organization; POS, point of service; PPO, preferred provider organization.

^a^ Statistics presented are all weighted values using the employer-sponsored insurance sampling weights.

^b^ Because of the large sample sizes, the SEs of proportion outcome measures were all close to zero; therefore, they were not reported.

^c^ The congestive heart failure sample and the diabetes sample do not include individuals with behavioral conditions examined in this study.

^d^ The Department of Health and Human Services HCC risk scores are designed similar to the Medicare HCC scores used in risk adjustment for Medicare Advantage plans; by design, the Department of Health and Human Services HCC scores are higher on average with wider distributions than the Centers for Medicare & Medicaid Services HCC scores. Our statistics are consistent with the literature.^17^

^e^ The total health care spending included all payments by insurance plans and cost-sharing payments from patients.

**Figure 1.**
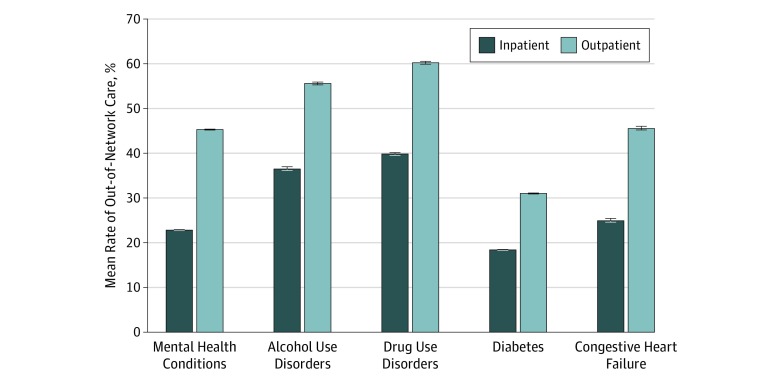
Mean Rates of Out-of-Network Care Among Individuals in the Top 25th Percentile of Total Health Care Spending Out-of-network care rates are weighted using sample weights to represent the national employer-sponsored insurance population. Rates were calculated conditional on individuals who incurred inpatient or outpatient care. The differences between behavioral conditions and congestive heart failure are statistically significant at the 95% CI for both inpatient and outpatient out-of-network care. The differences between behavioral conditions and diabetes are statistically significant at the 95% CI, for both inpatient and outpatient out-of-network care. The 95% CIs (error bars) were narrow because of the large sample sizes in our analyses.

As shown, the total annual spending amounts were highly skewed toward the top spenders. Because the top spenders accounted for most of the total spending within each group, we evaluated aggregated OON measures with a focus on the individuals in the top 25th and top 10th percentiles of annual total spending. Figure 1 addresses rates of OON care for those in the top 25th percentile of total spending. Encounters with OON providers were significantly more common among those with alcohol and drug use disorders in both inpatient and outpatient settings. For instance, 39.8% of those with drug use disorders whose total spending ranked in the top 25th percentile incurred cost-sharing payments for OON services during inpatient care. These rates increased to 60.3% for outpatient care. In contrast, for individuals with diabetes, OON care rates were 18.4% for inpatient care and 31.2% for outpatient care.

As shown in Table 2, individuals with CHF in the top 25th percentile of total spending had a mean of $147 727 (95% CI, $146 205-$149 249) in total health care expenditures, while those in the top 10th percentile had a mean of $259 919 (95% CI, $256 772-$263 066) in total health care spending. Those with diabetes had a mean of $45 315 (95% CI, $45 095-$45 535) in total health care expenditures for the top 25th percentile and a mean of $84 247 (95% CI, $83 764-$84 729) in total health care expenditures for the top 10th percentile. Mental health conditions, alcohol use disorders, and drug use disorders all had substantially less total spending than CHF. For example, individuals with mental health conditions in the top 10th percentile of total spending had a mean of $88 540 (95% CI, $88 166-$88 914), significantly less than that of CHF ($259 919) (*P* < .001).

**Table 2.** Aggregated Unadjusted Outcomes, According to Total Health Care Spending^a^

Outcome	Top 25th Percentile	Top 10th Percentile
Mean total health care spending, $ (95% CI)		
Mental health conditions	47 804 (47 634-47 973)	88 540 (88 166-88 914)
Drug use disorders	86 973 (86 218-87 728)	154 705 (153 137-156 273)
Alcohol use disorder	80 455 (79 743-81 167)	144 137 (142 655-145 619)
Congestive heart failure^b^	147 727 (146 205-149 249)	259 919 (256 772-263 066)
Diabetes^c^	45 315 (45 095-45 535)	84 247 (83 764-84 729)
Mean cost sharing for OON care (according to total health care spending percentiles), $ (95% CI)^d^		
Mental health conditions	1382 (1370-1394)	1750 (1725-1774)
Drug use disorders	2882 (2827-2936)	3725 (3618-3833)
Alcohol use disorder	2660 (2602-2718)	3543 (3430-3655)
Congestive heart failure^b^	1261 (1156-1366)	1740 (1514-1965)
Diabetes^c^	963 (939-986)	1245 (1199-1290)
Mean cost-sharing proportions for OON care (according to total health care spending percentiles), % (95% CI)^e^		
Mental health conditions	4.0 (4.0-4.1)	2.5 (2.5-2.5)
Drug use disorders	4.2 (4.1-4.3)	3.1 (3.0-3.1)
Alcohol use disorder	4.1 (4.0-4.1)	3.1 (3.0-3.2)
Congestive heart failure^b^	1.0 (0.9-1.0)	0.7 (0.6-0.7)
Diabetes^c^	2.6 (2.6-2.6)	1.7 (1.7-1.7)
Mean out-of-pocket proportions for OON care (according to total health care spending percentiles), % (95% CI)^f^		
Mental health conditions	25.3 (25.3-25.4)	25.5 (25.4-25.7)
Drug use disorders	35.9 (35.6-36.2)	40.3 (39.9-40.8)
Alcohol use disorder	34.0 (33.7-34.3)	39.1 (38.6-39.6)
Congestive heart failure^b^	17.9 (17.7-18.2)	20.3 (19.8-20.7)
Diabetes mellitus^c^	18.3 (18.1-18.4)	18.9 (18.7-19.0)

Abbreviation: OON, out-of-network.

^a^ Statistics presented are weighted values using the employer-sponsored insurance sampling weights. The outcome values are summarized, and the mean values within each percentile group were presented.

^b^ All values presented for individuals with mental health conditions, alcohol use disorders, and drug use disorders are statistically different from those of the congestive heart failure group at 95% CI.

^c^ All values presented for individuals with mental health conditions, alcohol use disorders, and drug use disorders are statistically different from those of the diabetes group at 95% CI.

^d^ Defined as cost-sharing payments associated with OON care, including copayments, coinsurance, and deductibles associated with OON clinicians and facilities.

^e^ Defined as cost sharing for OON care as a proportion of total health care spending. We presented the proportions as percentages.

^f^ Defined as cost sharing for OON care as a proportion of total cost sharing (ie, for both in-network and OON care). We presented the proportions as percentages.

Individuals with drug use disorders and those with alcohol use disorders in the top 25th percentile each spent a mean of more than $3500 for OON cost-sharing payments (drug use disorders, $3725 [95% CI, $3618-$3833]; alcohol use disorders, $3543 [95% CI, $3430-$3655]) (Table 2). However, despite substantially higher total health care spending for those with CHF, OON cost-sharing payments were a mean of $1261 (95% CI, $1156-$1366) for those in the top 25th percentile of total spending. The cost sharing for OON care among those with drug use disorders and alcohol use disorders accounted for over 30% of total cost-sharing payments for individuals in the top 25th percentile of total spending. In contrast, OON care accounted for less than 20% of total cost-sharing payments for both those with CHF and those with diabetes in the top 25th percentile of total spending.

Table 3 presents the estimated differences of outcome measures between individuals with behavioral health conditions and those with CHF or diabetes, after controlling for other characteristics.^14^ Each table cell represents results based on separate analyses. The probabilities of encountering OON providers in inpatient and outpatient care settings were both substantially higher for those with behavioral conditions. Specifically, drug use disorders were associated with higher probabilities of encountering OON providers in the inpatient care setting by 12.9 percentage points (95% CI, 12.5-13.2 percentage points; *P* < .001) and in the outpatient care setting by 15.3 percentage points (95% CI, 15.1-15.6 percentage points; *P* < .001) compared with CHF. Relative to CHF or diabetes, having behavioral conditions was associated with substantially larger cost sharing for OON care, as well as a higher portion of total out-of-pocket spending on OON care. For example, individuals with mental health conditions had cost-sharing payments for OON care $341 (95% CI, $331-$351) higher than those with diabetes (*P* < .001), individuals with drug use disorders had cost-sharing payments for OON care $1242 (95% CI, $1209-$1276) higher than those with diabetes (*P* < .001), and individuals with alcohol use disorders had cost-sharing payments for OON care $1138 (95% CI, $1101-$1174) higher than those with diabetes (*P* < .001).

**Table 3.** Differences in Estimated OON Care and Cost Sharing Between Behavioral Conditions and Chronic Conditions^a^^,^^b^

Characteristic	Marginal Effects (95% CI)				
	Cost Sharing for OON Care, $^c^	Cost-Sharing Proportion for OON Care, %^d^^,^^e^	Out-of-Pocket Proportion for OON Care, %^e^^,^^f^	Probability of OON Inpatient Care, %^e^	Probability of OON Outpatient Care, %^e^
**Relative to Individuals With CHF**					
Chronic mental health conditions	221 (188 to 255)^g^	2.2 (2.1 to 2.3)^g^	4.9 (4.6 to 5.1)^g^	4.2 (4.0 to 4.5)^g^	8.4 (8.2 to 8.5)^g^
Observations, No.	1 889 057	1 888 865	1 887 098	826 977	6 439 514
Drug use disorders	1033 (994 to 1071)^g^	1.2 (1.1 to 1.3)^g^	9.6 (9.3 to 9.9)^g^	12.9 (12.5 to 13.2)^g^	15.3 (15.1 to 15.6)^g^
Observations, No.	311 890	311 863	311 544	306 494	862 815
Alcohol use disorders	913 (873 to 953)^g^	0.9 (0.8 to 1.0)^g^	8.2 (8.0 to 8.5)^g^	11.2 (10.8 to 11.6)^g^	12.5 (12.3 to 12.8)^g^
Observations, No.	281 869	281 848	281 549	272 811	819 288
**Relative to Individuals With Diabetes**					
Chronic mental health conditions	341 (331 to 351)^g^	1.0 (0.9 to 1.0)^g^	5.7 (5.6 to 5.8)^g^	5.6 (5.4 to 5.8)^g^	13.1 (13.1 to 13.2)^g^
Observations, No.	2 446 049	2 445 796	2 443 042	1 023 398	9 571 008
Drug use disorders	1242 (1209 to 1276)^g^	0.3 (0.2 to 0.3)^g^	9.6 (9.4 to 9.7)^g^	14.6 (14.3 to 14.9)^g^	19.1 (18.9 to 19.2)^g^
Observations, No.	868 882	868 794	867 488	502 915	3 994 309
Alcohol use disorders	1138 (1101 to 1174)^g^	0.01 (–0.07 to 0.08)	8.3 (8.1 to 8.5)^g^	13.0 (12.7 to 13.3)^g^	17.0 (16.8 to 17.2)^g^
Observations, No.	838 861	838 779	837 493	469 232	3 950 782

Abbreviations: CHF, chronic heart failure; OON, out-of-network.

^a^ All analyses were performed conditional on receiving a certain type of care. For example, the estimation of cost sharing for OON medical care was conditional on having any OON care. Similarly, the probability of an OON encounter in inpatient or outpatient care was conditional on using inpatient or outpatient care, respectively.

^b^ Results reported in each cell were estimated using a separate equation. The estimates were adjusted for a list of covariates as explained in Methods.

^c^ Defined as cost-sharing payments associated with OON care, including copayments, coinsurance, and deductibles associated with OON clinicians and facilities. Results presented for cost-sharing OON medical care outcome were the marginal effets that captured the differences in adjusted dollar values between a behavioral group and a specific reference group.

^d^ Defined as cost-sharing amount for OON care divided by total health care spending.

^e^ Results presented for these outcomes are marginal effects that captured the percentage-point differences between outcomes of a behavioral group and outcomes of a specific reference group.

^f^ Defined as cost-sharing amount for OON care divided by total cost sharing (ie, for both in-network and OON care).

^g^ *P* < .001 at a 95% CI.

**Figure 2.**
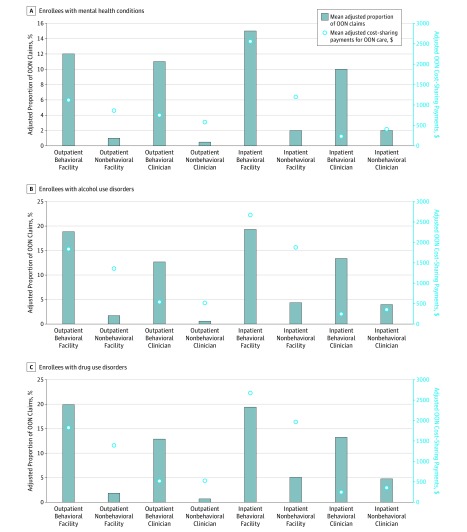
Out-of-Network (OON) Care and Cost Sharing Data represent results from regression analyses for each facility setting and clinician type as described in Methods. Left y-axis: mean adjusted proportion of OON claims. The outcome measure represents the proportion of claims that were OON. It is the OON claims for care at a facility or with a clinician divided by the sum of in-network and OON claims for care in that provider source. Right y-axis: mean adjusted OON cost sharing. The outcome measure represents cost-sharing payments for OON care at a facility or with a clinician.

Figure 2 shows the estimated proportions of OON care for those with behavioral conditions from behavioral and nonbehavioral providers. For example, for those with drug use disorders who had inpatient hospitalization claims at behavioral facilities, a mean of almost 20% were OON claims. Similarly high rates were observed for behavioral providers in both inpatient and outpatient settings. For nonbehavioral facilities, the proportion of claims that were OON was consistently much lower. Also, as shown in Figure 2 (right axis), cost-sharing payments for OON care specific for each provider source were substantial for behavioral providers. The summarized results from the regression analyses by provider source are included in the eTable in the Supplement. As shown by the numbers of patients for each provider type, overall care for those with behavioral health conditions is largely driven by nonbehavioral clinicians and facilities. Nonetheless, the likelihood of OON care was much higher for those who used behavioral sources of care.

## Discussion

Adults who have behavioral health conditions often face heavy financial burdens from health care spending.^23^ More than a decade ago, provisions in the Paul Wellstone and Pete Domenici Mental Health Parity and Addiction Equity Act of 2008 extended the parity of benefits to OON behavioral care providers to lessen patient financial burdens. However, our study found a striking disparity in cost sharing for OON care for privately insured adults with behavioral conditions compared with adults with other common chronic physical conditions. The observed differences appeared to be associated with higher rates of OON care and higher cost-sharing payments, not higher total health care spending. Those with behavioral conditions had substantially higher rates of OON care and cost-sharing payments when using behavioral care facilities or clinicians, compared with nonbehavioral health sources.

These findings underscore the potential challenges from narrow networks for behavioral clinicians and facilities for individuals with behavioral health conditions. Behavioral health clinicians have some of the lowest participation rates of all specialties, in both government-sponsored and private health plans.^7,24,25^ Combined with high demand and low rates of reimbursement, the workforce scarcity of behavioral health clinicians can represent the weakest component of health plan networks. For example, only about 62% of psychiatrists accepted new, privately insured patients.^8^ As a consequence, existing networks in commercial health plans may be too narrow to offer timely appointments, leading to the higher rates of OON care that we observed. In addition, patients with behavioral health conditions may continue existing relationships with selected clinicians even when they decline network participation.^6^ It is also possible that some enrollees in our study with behavioral health conditions consciously chose nonparticipating clinicians who did not participate in plan networks over perceived quality. Finally, some patients may not have ready access to in-network behavioral provider directories.

The disparities arising from the OON cost sharing also have different implications for care in outpatient and inpatient settings. For instance, in outpatient settings, patients most often schedule appointments for physician offices, clinics, or in hospital-based outpatient departments. Staff members in these settings conventionally ask patients about insurance coverage during the appointment process. However, for inpatient care, patients often present through the emergency department and have less discretion about choosing in-network clinicians. For even elective admissions to an in-network hospital, it would be unusual for patients to request, much less be informed about, the network status of specific clinicians who may be a part of the care team.

### Limitations

Our study has some limitations. First, the MarketScan database does not include details on specific benefit designs, such as deductible requirements or out-of-pocket maximums. Furthermore, we focused on OON care that was at least partially covered under ESI benefits. We did not assess uncovered out-of-pocket costs from care, nor did we observe additional sums that providers attempted to collect beyond health plan allowable reimbursements (“balance billing” practice). The data also cannot identify those who did not receive care because of cost barriers. Thus, our estimates of the cost-sharing burden for OON care were likely conservative. Nonetheless, our study demonstrates that cost sharing for even partially covered benefits for behavioral conditions can lead to high financial burdens for OON care.

In recent years, health care spending has shifted from employers to employees. Rapidly growing deductibles have particularly raised concerns—in 2018, the mean deductible for a single individual in an ESI plan reached $1573.^26^ Furthermore, enrollees typically face steeper cost-sharing provisions for OON care. In 2016, the mean deductible for in-network medical care was $1800 for an individual and $3900 for family coverage, while the mean deductible for OON coverage was $3000 for an individual and $6000 for family coverage. Similarly, the mean out-of-pocket annual maximum and coinsurance payments for OON care were nearly 2-fold those for in-network care.^12,27^ As a result, OON care can pose particularly difficult financial burdens for those with behavioral health conditions.

Our study could not address the market-based negotiations that take place between health plans and providers. Restricted networks are vital tools used by health plans to secure price discounts from participating providers to lower health care spending.^28^ However, health plans may seek to overleverage network participation for some providers, such as those specializing in behavioral health care. Conversely, providers may reject the low reimbursement from the health plan and remain OON as a business strategy. However, the consequence can be a potential inadequacy of provider networks, representing an access barrier to patients. Moreover, in the face of the escalating epidemic of drug use disorders, higher financial burdens caused by limited access to in-network care for people with mental health and substance use disorders are especially troubling. Employers need to balance the imperative to rein in health care spending with requisite access for patients. For the successful management and care of those with behavioral health conditions, a plan’s network should ensure timely and adequate access to in-network providers.

## Conclusions

The use of OON providers and associated cost-sharing payments were significantly higher for individuals with behavioral health conditions in employer-sponsored plans compared with those with other common chronic physical conditions. Cost sharing for OON care represents a substantial financial burden to patients with behavioral conditions, and it may be an important sign of network inadequacy that requires more scrutiny from policy makers.

## Notes

Supplement. **eAppendix.** Model Specifications **eReferences.** **eTable.** Average Adjusted Out-of-Network Care and Cost-Sharing Payments by Provider Type
