# Supplementary material for: Cost-Sharing Disparities for Out-of-Network Care for Adults With Behavioral Health Conditions
Source: JAMA Netw Open. 2019 Nov 6;2(11):e1914554. doi: 10.1001/jamanetworkopen.2019.14554 (PMC6865267; doi:10.1001/jamanetworkopen.2019.14554)
Supplement: Supplement. — eAppendix. Model Specifications eReferences. eTable. Average Adjusted Out-of-Network Care and Cost-Sharing Payments by Provider Type [file jamanetwopen-2-e1914554-s001.pdf]

Supplementary Online Content

Xu WY, Song C, Li Y, Retchin SM. Cost-sharing disparities for out-of-network care for adults with behavioral health conditions. *JAMA Netw Open.* 2019;2(11):e1914554. doi:10.1001/jamanetworkopen.2019.14554

eAppendix. Model Specifications
eReferences.
eTable. Average Adjusted Out-of-Network Care and Cost-Sharing Payments by Provider Type

This supplementary material has been provided by the authors to give readers additional information about their work.

© 2019 Xu WY et al. *JAMA Network Open.*

eAppendix. Model Specifications

The following represents model specifications that were used to estimate marginal effects displayed in Table 3, the eTable, and Figure 2.

In the analysis, unit of analysis is person-year. OON_{ijp} indicates the occurrence of any OON care associated with cost-sharing payments. A logit model is estimated to predict the probability of having any cost-sharing spending for OON care during a year. This model is estimated separately for inpatient care and outpatient care, and results are displayed in Table 3.

The model is specified as:

$$logit\ [Prob(OON_{ijp} = 1 | X_{ijp})] = \beta_0 + \beta_1 Condition_i + \beta_2 HCC_i + \beta_3 Rural_i + \alpha_p + \gamma_j + \varepsilon_{ijp} \quad \text{(e1)}$$

Here, i indicates individual, p indicates a Plan Type (a vector of dummy variables), j indicates State (a vector of dummy variables).

The binary variable $Condition_i$ equals to 1 if the individual has a behavioral condition (e.g. drug use disorder). $Condition_i$ equals to 0 if the individual is from "Diabetes" or "CHF" condition group (these are separate reference groups). The coefficient β_1 captures the marginal effects of having a behavioral condition on the probability of having OON care relative to individuals with diabetes or CHF, adjusted for several individual-level variables to account for differences that could influence spending and healthcare use.

Health status (HCC_i) is represented by a risk score following definitions of HHS for commercial

population. The algorithm to calculate HCC_i already incorporated age and gender, thus the demographic factors were not separately controlled in the modeling. $Rural_i$ is a binary variable and 1 indicates rural residency of an individual.

α_p captures the effect of Plan Type p, and γ_j represents the effect of $State_j$.

This model is estimated for each of the analytic samples.

Among those who had any OON medical care (that is, $OON_{ijp} = 1$), the expected amount of total OOP cost-sharing payments for OON care covered by insurance (OOP_OON_{ijp}) was estimated using a generalized linear regression model (GLM) with a log link and Gamma family distribution. We define Y as the dollar values of OON cost-sharing. The results were presented in Table 3.

The model is specified as:

$$\log[E(Y_{ijp})] = \beta_0 + \beta_1 Condition_i + \beta_2 HCC_i + \alpha_p + \beta_3 Rural_i + \gamma_j + \varepsilon_{ijp} \quad \text{(e2)}$$

Among those who had any OON medical care, another GLM model with binomial distribution and log link was used to estimate the outcome measures of cost-sharing proportion for OON care in total health care spending, and the cost-sharing proportion for OON care in total out-of-pocket costs. We define the Z as proportion outcome measures whose values were a fraction of 1 (between 0 and 1). The estimation modeling was suggested by Papke & Wooldridge (1996). The results were presented in Table 3.

$$\log[E(Z_{ijp})] = \beta_0 + \beta_1 Condition_i + \beta_2 HCC_i + \alpha_p + \beta_3 Rural_i + \gamma_j + \varepsilon_{ijp} \quad \text{(e3)}$$

© 2019 Xu WY et al. *JAMA Network Open.*

The results presented in the eTable and in Figure 2 followed similar modeling approaches. In particular, the analyses to estimate the proportion of OON claims for a provider source in total OON claims followed the same modeling approach in Equation (e3), conditional on utilization of a provider source. The cost-sharing amounts were estimated following the same approach in Equation (e2), conditional on use of OON care for a specific provider type.

© 2019 Xu WY et al. *JAMA Network Open.*

eReferences

Baum, C.F. 2008. Modeling proportions. Stata Journal 8: 299–303.

Papke, L. E. and J. Wooldridge. 1996. Econometric methods for fractional response variables with an application to 401(k) plan participation rates. Journal of Applied Econometrics 11: 619–632.

© 2019 Xu WY et al. *JAMA Network Open.*

**eTable.** Average Adjusted Out-of-Network Care and Cost-Sharing Payments by Provider Type[†] [‡]								
**Average Adjusted Proportion of Out-of-Network Care by Provider Type**								
	Outpatient Behavioral Care Facility	Outpatient Non-Behavioral Care Facility	Outpatient Behavioral Care Provider	Non-Behavioral Care Provider	Inpatient Behavioral Care Facility	Inpatient Non-Behavioral Care Facility	Inpatient Behavioral Care Provider	Inpatient Non-Behavioral Care Provider
Chronic mental health conditions	0.12	0.01	0.11	0.005	0.15	0.02	0.10	0.02
N	180,303	4,046,675	2,128,958	5,962,547	49,823	671,507	141,509	654,071
Drug use disorders	0.20	0.02	0.13	0.01	0.19	0.05	0.13	0.05
N	61,341	391,798	166,193	515,555	41,337	173,587	67,069	154,476
Alcohol use disorders	0.19	0.02	0.13	0.01	0.19	0.04	0.13	0.04
N	50,394	353,271	136,281	474,398	29,644	147,332	48,958	135,006
**Average Adjusted Cost-Sharing Payments for Out-Network-Care by Provider Type ($)**								
	Outpatient Behavioral Care Facility	Outpatient Non-Behavioral Care Facility	Outpatient Behavioral Care Provider	Outpatient Non-Behavioral Care Provider	Inpatient Behavioral Care Facility	Inpatient Non-Behavioral Care Facility	Inpatient Behavioral Care Provider	Inpatient Non-Behavioral Care Provider
Chronic mental health conditions	$1,117	$864	$748	$579	$2,558	$1,198	$234	$405
N	31,006	283,511	357,308	1,094,175	10,091	54,168	27,857	108,366
Drug use disorders	$1,825	$1,394	$519	$528	$2,676	$1,966	$243	$354
N	17,021	46,095	39,053	114,744	10,953	23,538	15,874	34,632
Alcohol use disorders	$1,837	$1,363	$542	$518	$2,672	$1,879	$246	$354

© 2019 Xu WY et al. *JAMA Network Open.*

N	13,298	37,314	30,732	97,522	7,972	17,893	11,549	28,953

† Results presented are based on GLM regression analyses as described in Methods.

A GLM model with log link and binomial family distribution was used to estimate the proportion of out-of-network care by provider type. This analysis is conditional on use of care in a provider type.

A GLM model with log link and gamma family was used to estimate the cost-sharing payments for out-network-care for each provider or facility type. This analysis is conditional on use of OON care in a provider type.

‡ The results presented were averaged estimated values.

© 2019 Xu WY et al. *JAMA Network Open.*
